# Chemical Mechanism of Allosteric and Asymmetric Dark Reversion in a Bacterial Phytochrome Uncovered by Cryo-EM

**DOI:** 10.1021/jacs.5c17531

**Published:** 2025-11-29

**Authors:** Szabolcs Bódizs, Anna-Lena M. Fischer, Miklós Cervenak, Sayan Prodhan, Michal Maj, Sebastian Westenhoff

**Affiliations:** † Department of Chemistry for Life Sciences, 8097 Uppsala University, Uppsala 75123, Sweden; ‡ Department of Chemistry—Angström, 8097 Uppsala University, Uppsala 75123, Sweden; § Science for Life Laboratory and Center for Chemical Mechanisms of Life, Department of Chemistry for Life Sciences, 8097 Uppsala University, Uppsala 75123, Sweden

## Abstract

Phytochromes are light-sensitive proteins that are found in plants, fungi, and bacteria. They exist in two functional states, Pr and Pfr, distinguished by *Z*/*E* isomers of their bilin chromophore. The chromophore can photoswitch between these states but also thermally converts in darkness. Despite the importance of the latter reaction, it is unknown how it is controlled by the phytochrome's structure. Here, we present single-particle cryo-EM measurements on the *Pseudomonas aeruginosa* bacteriophytochrome (PaBphP) carried out at multiple time points during dark reversion from Pr to Pfr. These experiments resolved the structure of a PrPfr hybrid state as a transient intermediate. Surprisingly, we find that only protomer B converts back to Pfr in the hybrid, while protomer A remains in Pr. We identify structural asymmetries in the precursor Pr state, which extend from the homodimer interface to a conserved histidine (H277). The hydrogen-bonding network around the chromophore is modulated, explaining how a phytochrome exerts control over the isomerization reaction. These findings establish that dark reversion is governed by conformational selection between two substates, whereby one is “dark-reversion ready” and the other blocks the reaction. Moreover, we explain how the equilibrium of the states is allosterically controlled across the dimer. Together, these findings provide a structural framework for tuning phytochrome signaling lifetimes in optogenetic applications.

## Graphical abstract

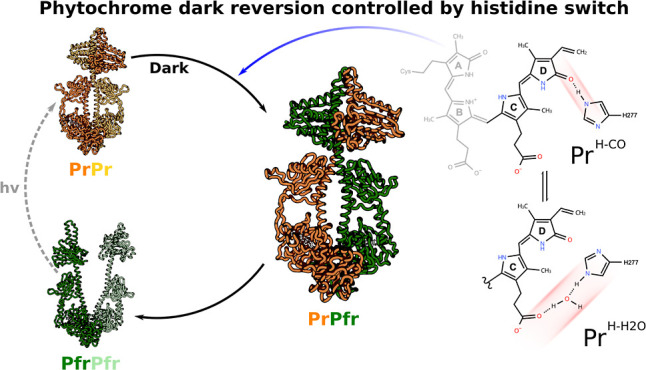

## Introduction

Phytochromes are universal and adaptable light sensors that regulate diverse biological processes across plants, bacteria, and fungi.^1–3^ These versatile photoreceptor proteins enable organisms to perceive and respond to light, temperature, and other environmental cues, influencing a wide array of physiological functions.^4–6^ In plants, phytochromes are central to developmental and stress response pathways, contributing significantly to fitness in natural environments and productivity in agriculture.^7–9^ Their mechanisms of action are diverse, involving changes in subcellular localization, protein–protein interactions, and crosstalk with other photoreceptors.^9–12^ In bacteria, they frequently act as histidine kinases in two-component signaling systems, although a variety of alternative output domains have evolved.^4,13^

Central to phytochrome function is their ability to reversibly switch between a red-light-absorbing Pr state and a far-red-light-absorbing Pfr state. This allows organisms to sense the ratio of red to far-red light in their environment. The photoswitching is initiated by light-driven *Z*/*E* isomerization of a covalently bound bilin chromophore within a conserved photosensory core module (PAS-GAF-PHY, see Figure 1b).^14^ Structural studies have revealed key conformational changes between Pr and Pfr, including isomerization of the chromophore, refolding of the “PHY-tongue” motif, and rearrangements of the quaternary structure.^15–20^ More recently, single-particle cryo-electron microscopy (cryo-EM) structures have started to provide insights into full-length phytochrome architecture in near-native conditions.^21–27^

**Figure 1:**
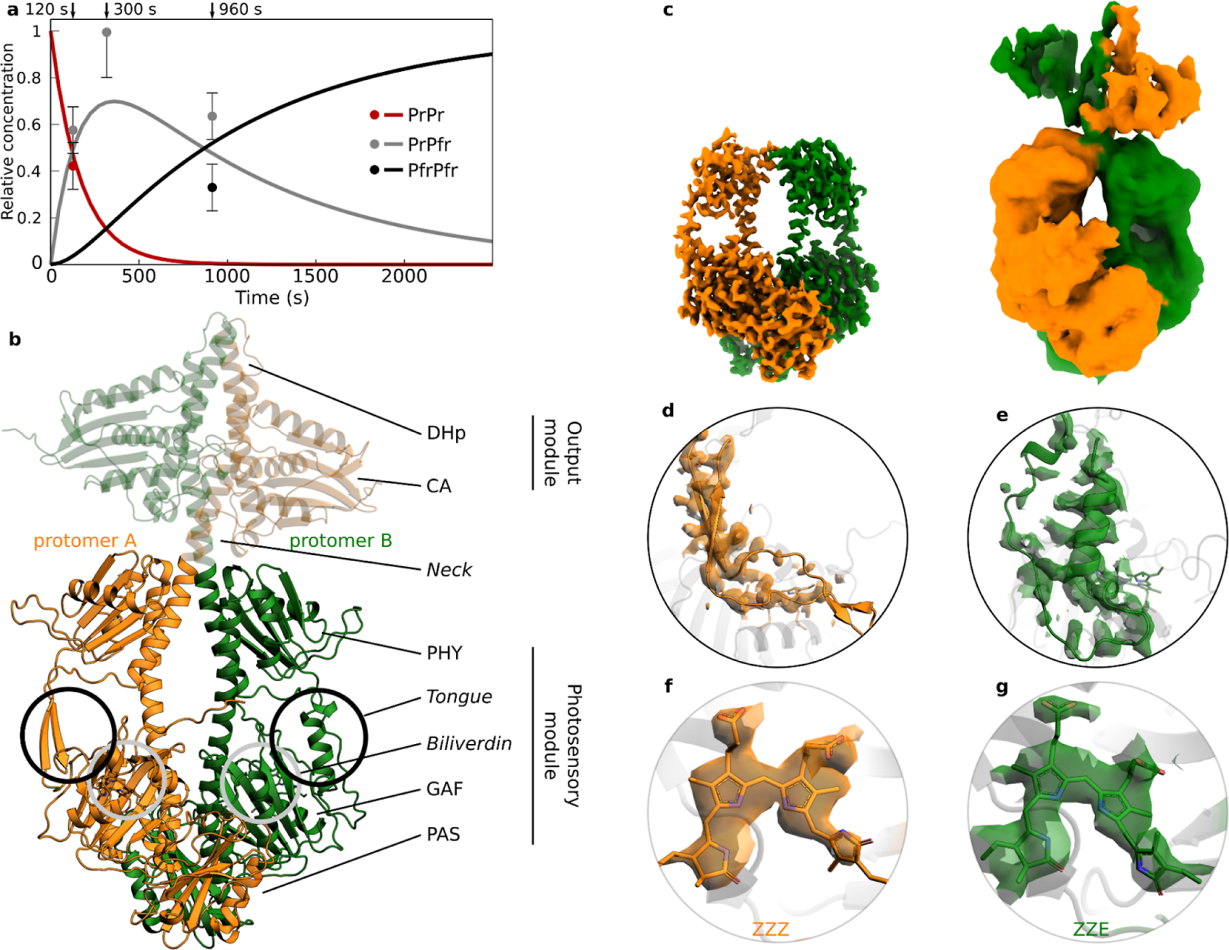
The dark reversion intermediate of PaBphP is a PrPfr hybrid. (a) The presence of a PrPfr intermediate state in the dark reversion reaction of PaBphP was revealed by UV–vis absorbance spectroscopy at 20 °C.^38^ Here, the time-dependent concentrations based on the rates identified by Prodhan et al. (2025) are shown. The time points at which cryo-EM data were recorded are indicated. The dots show the relative concentrations estimated from the number of particles assigned to each 3D volume in the reconstructions; the error bars are set to 0.1. At 300s the uncertainty was higher and the error bar for the PrPfr state was set to 0.2. PfrPfr particles were observed as 2D classes, and likely some PrPr particles are included in the 3D volume for the hybrid state, but neither of the homodimer states could be refined. (b) The cryo-EM structure of the PrPfr hybrid, reconstructed from the 300 s dark reversion data (the output module, not clearly resolved in the reconstruction, is not included in the final model but is shown here with decreased opacity). (c) The panel shows the coulomb density reconstructed via heterogeneous reconstruction of the full-length protein (right) and local refinement of the PSM (left). (d–g) The PHY-tongue (d,e) and the chromophore (f,g) show characteristic conformations for Pr-state in protomer a (d,f) and Pfr-state in protomer b (e,g).

In the absence of light, phytochromes return to their resting states. Most phytochromes dark-revert to the Pr state, although “bathy” bacterial phytochromes revert to Pfr.^28^ The dark reversion rates are species-dependent and modulated by pH, phosphorylation, and protein–protein interactions.^29–32^ Dark reversion plays a key functional role by determining the lifetime of the photoactivated state and thus tuning the organism’s sensitivity to ambient light cues. Evolution of paralogous genes allows plants to host several isoforms of phytochromes with different dark reversion rates, which, despite having partially similar spectral properties, can cater for a large dynamic range of signaling.^33^ Additionally, since the reversion rate is highly temperature-dependent, phytochromes can act as biological thermometers.^6,34,35^ Thus, the protein must be able to control the thermal reversion reaction of the bilin chromophore, but the structural and chemical bases for this remain poorly understood.

Phytochromes are typically dimeric proteins. This significantly enhances the signaling capabilities of the proteins, exemplified by the formation of PrPfr heterodimers, creating a distinct “third state” in addition to the PrPr and PfrPfr homodimers.^5,24,36^ In *Arabidopsis thaliana* PhyB the PrPfr heterodimer undergoes dark reversion approximately 100 times faster than the PfrPfr homodimer, which indicates strong intradimeric allostery and leads to effective suppression of PhyB-mediated signaling under low light intensity.^37^ While the existence of heterodimeric states has long been recognized in plants, evidence of PrPfr states in bacterial phytochromes is scarce. To date, only one crystal structure of an engineered bacterial phytochrome and a recent cryo-EM structure from a myxobacterial phytochrome have captured such hybrid forms.^20,24^ In our recent work using optical spectroscopy, we show that PrPfr heterodimeric states are indeed populated during dark reversion in bacterial phytochromes.^38^ Notably, we observed clear evidence of the allosteric regulation of the dark reversion rate.

Despite its physiological importance, dark reversion remains poorly understood at the structural and chemical levels. For thermal reversion, the chromophore has to isomerize around the 15–16 double bond (Figure S1), which is expected to have a substantial activation energy on the order of 100 kJ/mol.^39^ This corresponds to about 40 times the *kT* at 298 K, and thus, prohibitively slow rates would be expected. However, faster rates are observed, implying that chemical mechanisms must be at play to lower the activation energy. One proposed mechanism that fulfils this requirement is the keto–enol tautomerization on the D-ring of the biliverdin in its Pr form, which reduces the bond order of the C15–C16 bond and hence lowers the activation barrier for thermal reversion.^40–42^ In this model, the enol form has to be formed before *Z*-to-*E* isomerization of the biliverdin can occur. Quantum chemical calculations suggest that this is possible when water molecules participate in the transition state between keto and enol forms.^41^ Moreover, pH-dependent spectroscopy suggests that the concentration of the enol state correlates with the protonation states of a histidine in the vicinity of the chromophore.^43^

From these considerations, the question arises as to how phytochromes exert control over the dark reversion reaction, both in terms of interdimer allostery and with respect to the dark reversion mechanism itself. A realistic candidate is conformational selection, where two or more substates coexist in the protein, with differential propensity for dark reversion. Detection of the structure of such substates and particularly those with low abundance is a major challenge.^44^ Crystal structures of phytochromes are unlikely to capture such states because they are frozen out at low temperatures and in the crystal lattice. Nuclear magnetic resonance (NMR) and spectroscopic studies have indicated the presence of multiple ground state conformations of phytochromes in solution,^45–48^ but lack structural specificity. Thus, the conformational selection mechanism remains unproven, and a structural understanding of how the dark reversion reaction is controlled is still lacking.

Here, we use single-particle cryo-EM to determine the structure of the bathy phytochrome from *Pseudomonas aeruginosa* at several time points during the dark reversion. Intriguingly, we observe that the dark reversion is strongly asymmetric, where only one protomer reverts to form a hybrid PrPfr state. This raises the opportunity for identifying the structural mechanisms with which the thermal isomerization of the biliverdin is controlled from within the same phytochrome.

## Results

### PrPfr Heterodimers Are Confirmed as Dark Reversion Intermediates

In order to solve the structure of the PrPfr heterodimer state during dark reversion of PaBphP, we made use of its expected relative concentration as derived from spectroscopic observation (Figure 1a),^38^ and cryo-trapped the protein on electron microscopy grids at 120 s, 300 and 960 s after photoexcitation with a laser diode at 780 nm. At 120 s, a new structural state (Figure 1b,c) is observed in the protein ensemble (approximately 58% of the particles) along the known PrPr structure (see Figure S2 for details of the reconstruction).^25^ At 300 s, the new state dominates the ensemble of the grids. Neither the homodimeric PrPr nor the PfrPfr states could be separately reconstructed, but a small number of open, PfrPfr-like particles can be identified during 2D classification (Figure S3). At 960 s, a mixture of the new state (approximately 64% of all particles) and the open PfrPfr state (approximately 36%) is present (Figure S4).

The newly observed structural state is a PrPfr heterodimer (Figure 1b) with one protomer in Pr and the other one in Pfr, as judged based on the secondary structure of the PHY-tongue (Figure 1d,e), isomerization state, and location of the chromophore (Figure 1f,g), and the rotamerization states of key residues lining the binding pocket (Figure S5). The structure of the PrPfr heterodimer was highly similar at all three time points and was, next to the already known PrPr and PfrPfr states, the only structural state identified from the particle ensemble at any of the time points.^25^ It is difficult to determine the fractional population of the three states quantitatively from the analysis of the cryo-EM data; however, the estimated concentrations agree well with the kinetic prediction (Figure 1a). This confirms that the spectroscopic intermediate indeed is a PrPfr heterodimer.

### Two Geometries at the Dimer Interface Resolved for PrPfr and PrPr States

The observed PrPfr heterodimer structure is naturally asymmetric at the tongue and chromophore regions due to the different photochromic states. However, we also observe strong asymmetry beyond these regions at the dimer interface of the PAS/GAF domains. Here, two distinct geometries are observed. In geometry I (Figure 2d), consisting of the GAF_A_ and PAS_B_ domains (subscripts denoting the protomer), helix A_A_ is in a conformation consistent with the Pfr state crystal structure, making contact with spine_B_, but not connecting to PAS_B_. This results in an open interface (Figure 2b,d).^17^ Geometry II (Figure 2e) involves GAF_B_ and PAS_A_ and here helixA_B_ is oriented into a 3-helix bundle with helix B_B_, spine_B_, and makes direct contact with PAS_A_. This results in a closed interface (Figure 2b,e). Highly similar geometries are also observed for the PrPr state and a characteristic gap between GAF_A_ and PAS_B_ (see arrow) is observed in both PrPr and PfrPr (Figure 2a).^25^ Figure 2c–e visualizes how the interaction network of helix A changes between the two interface geometries. Several key interactions change, affecting the neighboring spine helix, helix B, and the connection to residues in the PAS domain. We conclude that the interface region at the PAS-GAF domains is intrinsically asymmetric in PrPr and PrPfr (but not in PfrPfr)^25^ and that the positioning of helix A plays a decisive role in breaking the symmetry. During reconstruction of the cryo-EM maps, this asymmetric dimer arrangement drives alignment, resulting in high-confidence assignments for protomers A and B in the PrPr and PrPfr states.

**Figure 2:**
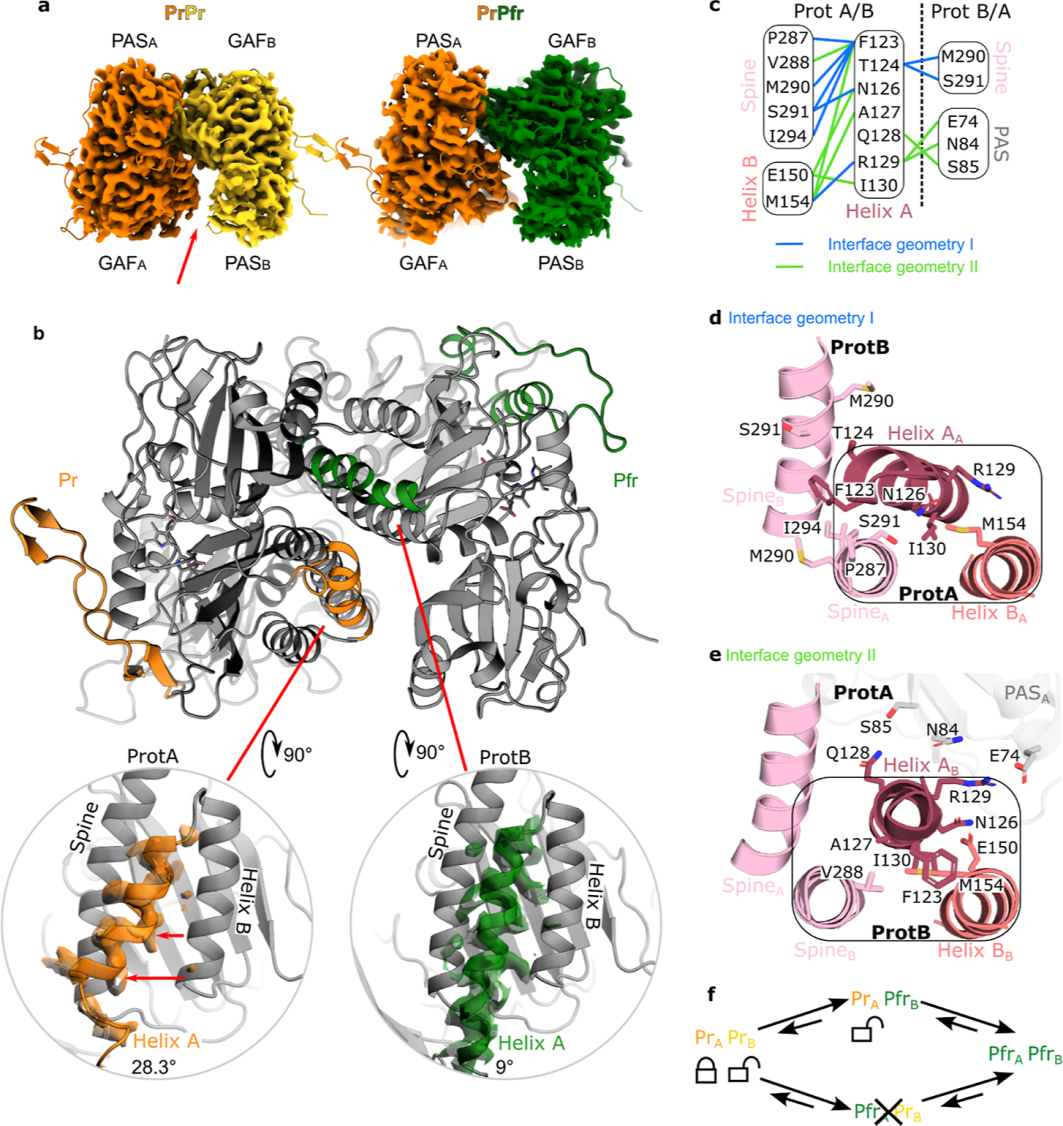
The PSM dimer interface is asymmetric in both the PrPr ground state and the PrPfr intermediate. (a) Two distinct PAS-GAF interaction geometries are observed in the PrPr and the hybrid PrPfr states, conserved during the transition from one to the other (red arrow points at the gap related to geometry I). (b) The orientation of helix A differs between the two protomers and correlates strongly with the photochromic state of the protomer in PrPfr: the relaxed helix A, the α-helical PHY tongue, and ZZE state chromophore are all exclusive to protomer B (green). This implies a strongly asymmetric dark reversion reaction, in which only protomer B dark converts from PrPr to PrPfr. The angle in the insets is calculated between helix A (residues 123–136) and the base of the spine helix (residues 286–300). (c) Distinct interface geometries according to interactions (interaction frequency differences) for each helix A of PrPr are shown schematically. Geometry I (blue) shows interactions of ProtB and geometry II (green) shows interactions of ProtA. Structural representations highlight the shift from interactions of helix A_A_ to Spine_B_ (d) to interactions of Helix A_B_ with PAS_A_ (e). (f) Schematic of the observed dark reversion sequence: the ProtA-Pfr hybrid state was not found in the cryo-EM data and was determined to occur less frequently in the MD simulations.

### Only One Protomer of the Asymmetric Dimer Dark-Reverts from PrPr to the PrPfr Hybrid State

Since the asymmetric arrangement of the PAS/GAF dimer interface is retained between PrPr and PrPfr, it provides a handle to trace protomers A and B separately in their dark reversion process. If the dark reversion was stochastic between the protomers, either protomer A or B could back-convert, and blurred densities would be expected at the chromophore and tongue positions because the two conformations would be averaged over each other. However, this is not observed. Instead, we recover high-resolution information at the chromophore and tongue in both protomers, which allows clear assignment to each one of the photochromic states (Figure 1 d–g). This leads to the surprising observation that only one of the protomers (protomer B) dark-reverts from the PrPr state to the hybrid PrPfr state (Figure 2f). We conclude that the dark reversion reaction in protomer A must be very slow or inhibited. It seems very likely that the difference in reactivity is caused by the observed structural asymmetry of the dimer in PrPr.

### A Switch of H277 and Its Hydrogen Bonding to the Chromophore Explains How Dark Reversion Is Controlled

Control over dark reversion requires regulation of the isomerization reaction at the biliverdin. In order to identify this mechanism, we reanalyzed our previously published electron density map (EMD-19981) and model (PDB 9EUT) of the PrPr state.^25^ Figure 3a,c shows that the density around the biliverdin in protomer B is weaker than in protomer A, which indicates a higher degree of heterogeneity in protomer B compared to A. Moreover, there is a difference in the conformation of the conserved histidine 277 (H277) with respect to the biliverdin: in protomer A the distance of the carbonyl oxygen of ring D of the BV (OD) to the epsilon nitrogen on the histidine (NE) is 2.6 Å with an angle of 150°, which is a suitable hydrogen bonding geometry; while in protomer B this distance is increased to 2.9 Å with an angle of 110°, which is not well compatible with hydrogen bonding in protomer B. Local resolution of the maps around the chromophore is ∼2.4 Å, which allows for confident placement of the histidine side chains.^25^ Additionally, the maps provide clear support for the refined positions of H277 as its electron density extends further toward the BV in protomer A compared to B (Figure 3a,c). Further down we will extend the structural analysis and find that the cryo-EM densities likely represent a mixture of substates. It is intriguing that the structural observations correlate with the ability of the protomers to react back to Pfr and suggests that, when hydrogen bonding of H277 to the D-ring carbonyl is direct as in protomer A, the dark reversion reaction becomes inhibited.

**Figure 3:**
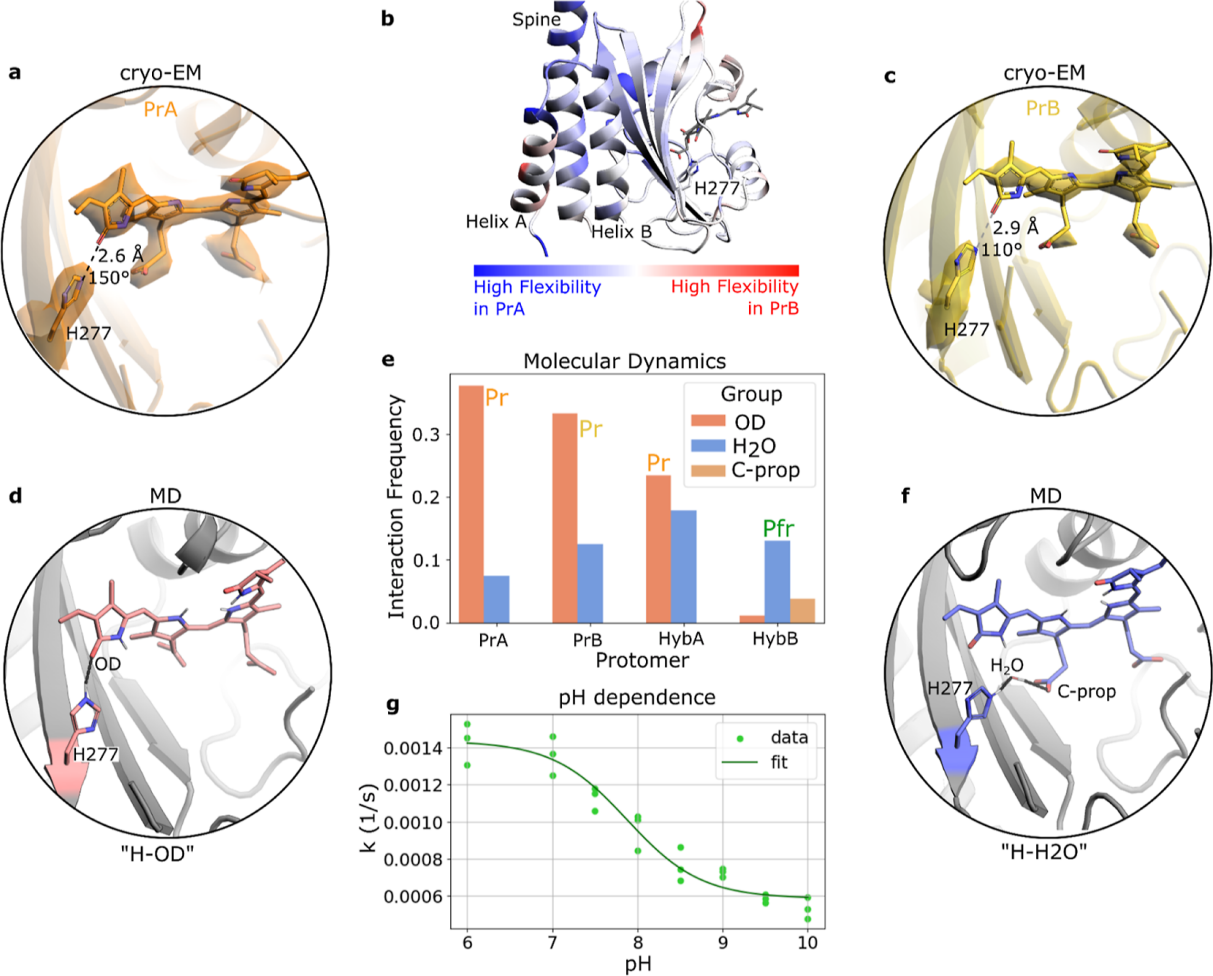
Distinct interaction pattern of H277 with BV reveals back-reversion advantage of protomer B. (a,c) Densities of biliverdin at identical contour levels in protomers of PrPr with distances from H277-NE to BV-OD and angles from H277-NE via H277-H_NE_ to BV-OD. (b) The difference in RMSF of the two protomers (which is comparable to an experimental *B*-factor and a measure for residue-wise flexibility) highlights a rigidification in protomer B around the active site. (e) Polar contact (hydrogen bonds) frequency of the different protomers where H277 interacts with biliverdin (H277-OD, “H-OD”) directly or via bridging waters (H277–H2O-Cprop, “H–H2O”) in each protomer of PrPr (PrA and PrB) and PrPfr (HybA and HybB). (d,f) Structural evaluation of the different states of the binding pocket: (d) H277-OD and (f) H277–H2O-Cprop. OD is the carbonyl of the D-ring and C-prop describes the carboxylates of the C-propionate. (g) pH dependence of dark reversion kinetics (rate constant vs pH) shows p*K*_a_ of 7.9. For additional details, see Figure S9.

To further elucidate the dynamics of biliverdin and H277, we performed three repetitions of molecular dynamics simulations (MD) for both the PrPr and PrPfr states. Within the 250 ns simulation time, the interface geometries predominantly stayed constant (Figure S6). Using these trajectories, we performed an interaction analysis, where the frequency of contacts between residues is determined according to geometric and electrostatic cutoffs. Higher interaction frequencies thereby indicate higher stability of the state. We find two predominant conformations, where H277 either forms a hydrogen bond directly to the D-ring (pattern “H-OD”, Figures 3d and Supporting Information) or it forms a bond via a water molecule to the propionate group of ring C (pattern “H–H2O”, Figure 3f). The switch is manifested by a retracting and twisting movement of the imidazole ring of H277, which leads to the change of hydrogen bonding. The fact that the trajectories sample both conformations in all of the MD trajectories implies that the states are close in energy and that they can interconvert within nanoseconds (Figure S7). The switch in hydrogen bonding of H277 has a direct effect on the conformation of the D-ring, which twists around V5 (torsion between C and D ring, NC–C14–C15–C16, see numbering in the Supporting Information) by 7.1° (Figure S8).

Figure 3e summarizes the frequencies with which each of the two predominant conformations occurs in the simulations. For the PrPr state, we find that the “H-OD” pattern is prevalent in protomer A (“H-OD” ∼ 38%, “H–H2O” ∼ 7%). In protomer B, the occurrence of the “H–H2O” increases while “H-OD” decreases (“H-OD” ∼ 33%, “H–H2O” ∼ 12%). This suggests that hydrogen bonding between H277 and the D-ring is tighter in the nonreactive protomer A. While the absolute frequencies of these patterns from MD trajectories may not be 100% reliable because of computational limitations (i.e., imperfect force field and water model), the increased “H–H2O” interactions observed between the two otherwise highly similar protomers support the robustness of the observed trend. The observation suggests that regulation of the dark reversion reaction occurs by control of the abundance of the reversion-ready “H–H2O” conformation, where H277 is not bound to the D-ring. Importantly, this conclusion aligns with the observations from cryo-EM maps (Figure 3a,c).

Turning to the MD simulations of the hybrid PrPfr state, we intriguingly find that the “H–H2O” conformation of H277 in protomer A increases in frequency compared to PrPr (“H-OD” ∼ 23%, “H–H2O” ∼ 18%). This finding is supported by the cryo-EM structures of the hybrid state, where the H277 in protomer A of PrPfr is refined into a position with distance of 2.6 Å and 120° with respect to the D-ring carbonyl, which is a conformation that resembles that of protomer B in PrPr and is unfavorable for direct hydrogen bonding. This suggests that protomer A can now undergo dark reversion from PrPfr to PfrPfr (Figure 3e).

### pH-Dependent Dark Reversion Rates Support the Role of a Histidine Residue in Controlling Chromophore Isomerization

To experimentally probe the proposed role of the histidine residue in the dark reversion process, we measured the pH dependence of the dark reaction kinetics (Figures 3g,S9 for additional information). The data revealed a sigmoidal decrease in rate constants with rising pH between values of 6 and 9, with an apparent transition midpoint near pH 7.9. The type of pH profile indicates that a single titratable group controls the reaction kinetics. These results are consistent with H277 regulating biliverdin isomerization and support the interpretation of the cryo-EM data and MD simulations.

### Protein Dynamics Link the H277 Switch to Conformation of the Dimer Interface

After highlighting the importance of the positioning of H277 for dark reversion (Figure 3) it is further relevant to elucidate how the asymmetry at the dimer interface and, in particular, the positioning of helix A (Figure 2) are connected to this molecular switch. Inspection of the MD trajectories and the cryo-EM structures did not reveal a clear pathway of concerted structural changes. Instead, we find correlated differences in the dynamics as modeled by the MD simulations. In protomer A, the spine helix, helix B, and the five-membered beta-sheet of the PAS domain, which connects to H277, are notably more flexible than those in protomer B according to the RMSF (Figure 3b). Only helix A is more rigid in protomer A, pointing at a stabilization due to the formed interactions with spine_B_. The observation suggests that the asymmetric arrangement of helix A at the interface (geometry I, Figure 2d) and the reactivity of the biliverdin are connected via altered dynamics of the PAS/GAF domains. Rigidification of the active site enables stabilization of the surrounding residues and thus opens up the space for H277 to retract from direct hydrogen bonding with biliverdin.

In conclusion, we find two patterns of hydrogen bonding of H277, which correlate with the dark reversion propensities of the two protomers. This leads to strongly asymmetric dark reversion: Thermal isomerization of biliverdin in protomer A is blocked or prohibitively slow in PrPr, which corresponds to the dominant occurrence of hydrogen bonding of H277 directly to the D-ring. However, in protomer B, the biliverdin is not hydrogen bonded to H277 and the reversion reaction occurs. In the PrPfr heterodimer state, the hydrogen bond pattern of protomer A is altered, with H277 binding much more frequently in the “H–H2O” arrangement, thus freeing up biliverdin for thermal isomerization. The cryo-EM structures show that this molecular switch of H277 is correlated with the asymmetric structure at the dimer interface, and the MD simulations suggest that the signal is transduced by modulation of the internal dynamics in the PAS-GAF domains.

## Discussion

### The PrPfr Intermediate Solved from Dark Reversion May Be Functionally Important

PrPfr intermediates in the phytochrome photocycle have been described in plants and, more recently, in bacterial systems.^5,20,24,36,37^ Here, we demonstrate that the PrPfr state is a key intermediate in the dark reversion reaction of bacterial phytochromes, structurally confirming our recent spectroscopic assignment.^38^ In plants, it is established that the intermediates are functionally relevant, enhancing the signaling potential of the protein.^37^ Thus, even bacterial phytochromes may use the PrPfr hybrid states for signaling. The role of PaBphP in its organism is to initiate biofilm formation in darkness when the bacteria enter the body.^49^ This is consistent with the comparatively fast dark reversion time, but it is not obvious how allosteric regulation or the presence of a hybrid state would be beneficial for this. We note, however, that state-of-the-art functional assays have not explicitly considered the possibility of a transient hybrid state for PaBphP and other bacterial phytochromes, thus it could be that the function of the PrPfr hybrids have so far eluded detection.^50,51^

### The PAS-GAF Dimer Interface Is an Important and Previously Unrecognized Regulatory Site

A large number of structural elements, including the chromophore, its direct environment, the PHY-tongue, and the output domains, have been implicated in the photo- and dark reactions of phytochromes.^15–20,42,52^ In this work we add the dimer interface at the PAS-GAF domains to this group and find that the distinct conformations of helix A in the two protomers strongly correlate with the ability of the connected chromophore to undergo dark isomerization. Notably, photoinduced changes in the interface region were also observed in an NMR investigation of a monomeric variant of the *D. radiodurans* phytochrome and in the cryo-EM structures of the PrPr, PrPfr, and PfrPfr states of the canonical bacteriophytochrome SaBphP2.^24,53^ In the latter study, the PrPfr heterodimer had an asymmetric position of helix A (Figure S10) and it is conceivable that this allosterically controls the rate constants of reversion in SaBphP2, in analogy to the findings made here for PaBphP.

### The Histidine Switch Works Together with Keto–enol Tautomerization as a Key Regulator in the Dark Reversion Process

Importantly, our structural data suggest a conformational selection mechanism for phytochrome dark reversion, in which substates of H277 are in equilibrium, and their relative abundance regulates the reaction kinetics (Figure 4). In the PrPr state, H277 adopts two distinct conformations. In the first one, it forms a direct hydrogen bond with the D-ring carbonyl, whereas in the other one, it connects to the C-propionate group via a water molecule (Figure 4b). The relative abundance of the two states is allosterically controlled by the symmetry break at the dimer interface (Figure 4c) and dynamics in the PAS/GAF domains linking the dimer interface and H277 (Figure 4a). These changes are small in amplitude, but are large enough to alter the hydrogen bonding environment of the D-ring, thereby resulting in large effects on dark reversion rates.

**Figure 4:**
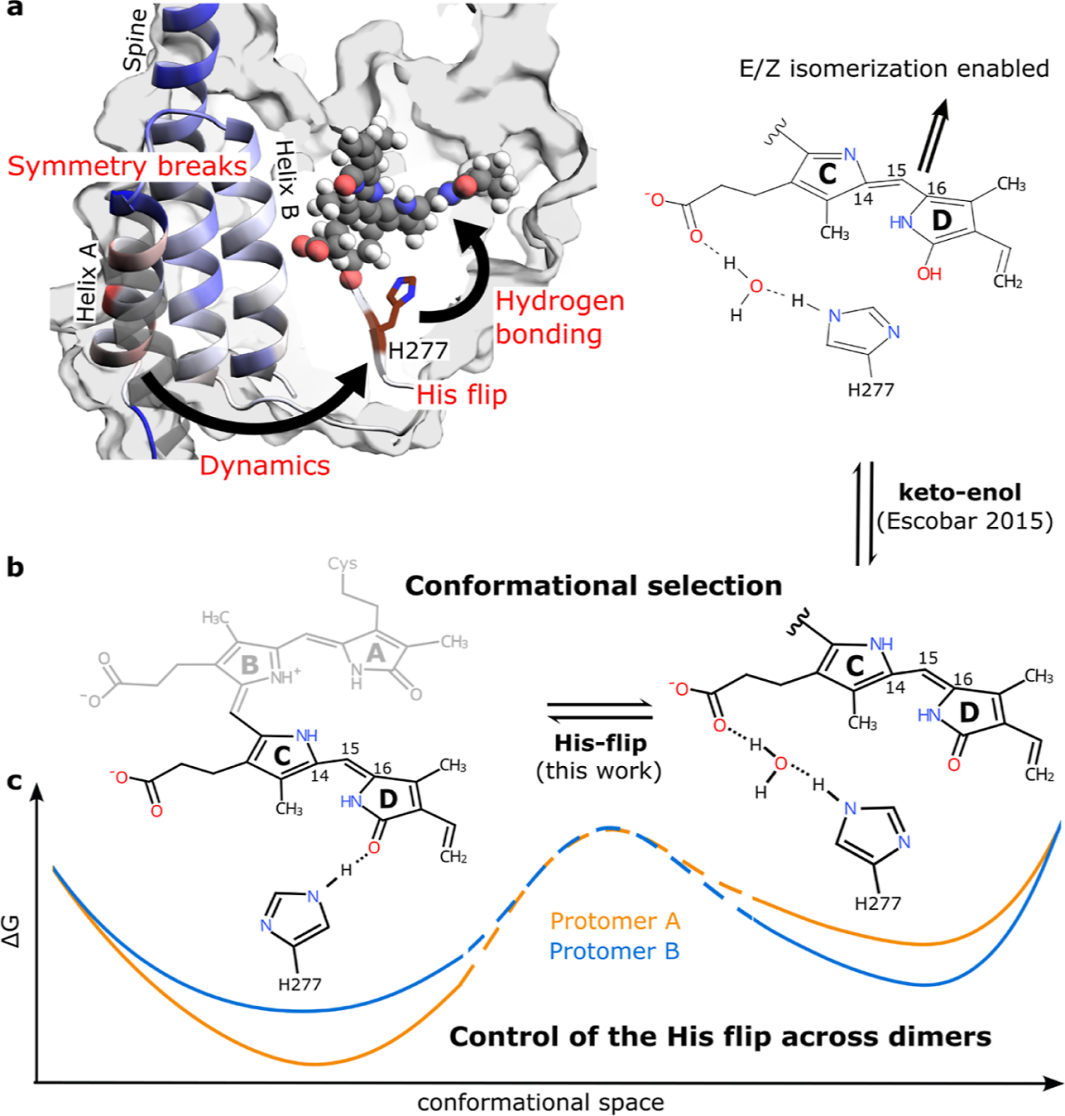
Summary of the asymmetric dark reversion mechanism. (a) Close-up of a phytochrome PAS-GAF domain shows the influence of the asymmetric interface (Helix A) on H277 (dynamics) and BV (hydrogen bonding). The helices are colored according to the difference in RMSF of the two protomers (blue means protomer A is more flexible) and the alternative conformation of helix A is shown in gray. (b) Combining the keto–enol tautomerization idea by Velazquez Escobar et al.^42^ with the difference in hydrogen bonding of H277 described in this work and highlighting the importance of conformational selection for the mechanism. (c) The schematic free energy landscapes correspond to the observed shifts in abundance of the different substates. Protomer A prefers the “H-OD” conformation (left) and protomer B the “H–H2O” conformation (right) when in Pr. Considering the higher occurrence of “H–H2O” in protomer B, from which dark reversion reaction can occur, this is in line with protomer B dark reverting first, coherent with the observations from the cryo-EM structure of the PrPfr hybrid state.

The switch of H277 identified here works in concert with the suggested keto–enol tautomerization mechanism of dark reversion in bathy phytochromes.^42^ In this mechanism, internal proton transfer from the B-rings to the D-ring carbonyl reduces the double-bond character between C15 and C16, thereby lowering the barrier for ring rotation. When H277 binds directly to the D-ring carbonyl, it stabilizes the keto form and inhibits tautomerization into the enol form (Figure 4b), thereby effectively blocking isomerization. When H277 switches to its alternative conformation and binds to the C-ring propionate via a water molecule, the carbonyl is freed from hydrogen bonding and may transition into the enol form, and dark reversion can proceed. Our (Figure 3g) and previously measured pH dependencies of the dark reversion further confirm the balance-tipping role of a histidine for the dark reversion kinetics.^42,54^ Moreover, the proposed mechanism is in line with quantum chemical computations of the keto–enol reaction, which emphasize the importance of water molecules for access to the enol as they stabilize the transition state between the two forms.^41^ This is fulfilled for the conformation where H277 does not bind to the D-ring C=O.

### Cryo-EM Is a Promising Technique for Measuring Conformational Heterogeneity

As a single-molecule technique, cryo-EM is in principle well suited to measure conformational heterogeneity such as that detected here. However, it still struggles to reach the resolution needed to resolve chemically relevant differences in small and flexible complexes as the conformational changes we detect in our system are too small in amplitude to be sorted from the data. The detection of low-abundance, high-energy states has so far been driven mainly by NMR, but our work shows the way for cryo-EM to achieve the same.^44,55^ In the present case, we had to complement the structural data with molecular dynamics simulations, but future methodological advances may enable cryo-EM to characterize such states more autonomously.

## Conclusion

Here, we have shown that conserved H277 and the surrounding hydrogen-bonding network play a key role in regulating the dark reversion reaction of phytochromes. H277 is widely conserved across the phytochrome family and has been shown to alter the isomerization yield of the Pr-to-Pfr photoreaction.^28,56–58^ The mechanism is also in general agreement with the detection of structural heterogeneity for phytochromes from NMR and time-resolved optical spectroscopy experiments.^45–48^ Allosteric control of dark reversion rates is a common feature of phytochromes.^36,59^ Here, we show that a break in symmetry at the dimer interface, linked to a change in the hydrogen bonding of H277, is a key chemical factor controlling this process. We hope that this conformational selection mechanism of the isomerization reaction can be used for the design of future optogenetic phytochrome variants.

## Methods

### Protein Purification

PaBphP was recombinantly expressed in *E. coli* BL21 cells transformed with the PaBphP gene (GenBank: AAG07504.1, on a pET28a­(+) backbone) and a heme oxygenase gene kindly provided by Prof. Janne A. Ihalainen. The cells were grown in LB media containing 50 μg/mL kanamycin and 34.5 μg/mL chloramphenicol at 37 °C until OD_600_ reached 0.6, then supplemented with 1 mM IPTG and 1 mM 5-aminolevulinic acid before lowering the incubation temperature to 18 °C.

The cells were processed in lysis buffer (50 mM Tris, 150 mM NaCl, 10% glycerol, 10 U/mL of DNase 1 and one tablet of complete EDTA-free protease inhibitor (Roche) at pH 8.0) using an EmulsiFlex C3 homogenizer (Avestin), and the lysate briefly incubated with a molar excess of biliverdin hydrochloride. The protein was then purified through a two-step chromatography protocol: first, Ni-NTA affinity chromatography (HisTrap HP 5 mL, Cytiva) by washing the bound sample with wash buffer (50 mM Tris and 1 M NaCl at pH 8.0) and eluting with elution buffer (50 mM Tris, 50 mM NaCl, 300 mM imidazole at pH 8.0) followed by size-exclusion chromatography (HiLoad 16/600 Superdex 200 pg, Cytiva) in SEC buffer (30 mM Tris at pH 8.0). The purified protein was collected at a concentration of 3 mg/mL and flash-frozen for storage.

### Cryo-Electron Microscopy Specimen Preparation

For structural studies, the protein was diluted to 1.5 mg/mL and the buffer exchanged to 80 mM Tris, 10 mM MgCl2 and 150 mM CH3CO2K at pH of 7.8. The bulk sample was illuminated with a 780 nm light source over 30 s (total dose: ∼1 mJ/mm^2^), then left in complete darkness until deposition on glow discharged UltrAuFoil (Quantifoil) R 1.2/1.3 (300 mesh) grids and flash-freezing in liquid ethane. The dark reversion time was measured from the removal of the far-red light source and was considered to be terminated at the moment of flash freezing. Specimens with 120, 300, and 960 s of dark reversion were prepared. The photon dose is sufficient to achieve full conversion into Pr.

### Cryo-Electron Microscopy Data Collection

The specimens were imaged using a Titan Krios G2 (Thermo Scientific) 300 keV transmission electron microscope equipped with a K3 (Gatan) detector and a BioQuantum (Gatan) energy filter. The data acquisition settings were as follows: 0.828 Å/px (105kx magnification), 50 e/Å^2^ total electron dose, −0.8––2.0 mm defocus range, and 20 eV energy filter slit width. Automated collection was performed using the EPU software (Thermo Scientific). A total of 13,534 movies were collected for the 120 s time point, 14,573 movies for the 300 s time point, and 13,112 movies for the 960 s time point.

### Cryo-Electron Microscopy Data Processing

The collected micrographs were processed using Cryosparc v4.6.2. Preprocessing consisted of patch motion correction, patch CTF estimation, and micrograph denoising (only for visualization purposes). A total of 13,393, 14,033, and 10,323 high-quality micrographs were accepted based on CTF fit, average defocus, and relative ice thickness. Particles were originally picked as 80–180 Å blobs. For the 300 s data set, a Topaz model was trained for more accurate picking, whereas in the other data sets the originally picked particle sets were used for further refinement. Removal of junk particles was done by subsequent rounds of 2D classification and heterogeneous refinement using both the open and closed dimers and several “junk” volumes as references.

For the data set at 300 s delay, a total of 964,467 particles were identified, which contributed to a PrPfr reconstruction. Further 3D classification did not reveal a separate PrPr class, but it was used to further select 488,190 particles with especially well-ordered α-helices in the PHY_B_ region (Figure S3). The failure to achieve a PrPr reconstruction is not proof of its absence in the population but indicative of bias toward the more populated PrPfr state during reconstruction. To account for this increased uncertainty at this time point, we increased the error estimate to 0.2 in Figure 1. With the 488,190 particle subset, separate local refinements were conducted with masks for the PAS-GAF-PHY regions of protomers A and B, which yielded maps of global resolution 2.87 and 2.85 Å, respectively. The output module was resolved at a nominal resolution of 5.4 Å. An atomic model was built using the ISOLDE (v1.6) extension of ChimeraX (v1.8), with the models 9EUY and 9EUT used as templates.^25,60^ The two locally refined maps were then combined using the Phenix function “Combine focused maps”.

For the data set at 120 s delay, a total of 895,611 particles were found. These were subjected to 3D classification using the “simple” initialization mode to reveal reconstructions of both the PrPr and the PrPfr states, followed by a second, targeted 3D classification round. 376,742 (42.1%) of the particles contributed to the PrPr map, while 513,575 particles (57.9%) yielded a PrPfr volume (Figure S2). Local refinements of the PAS-GAF-PHY regions (excluding the output module) were conducted on both classes, resulting in global resolutions of 3.02 and 2.82 Å, respectively. Comparing the two maps against 9EUT and the newly built PrPfr models showed good fits, so no new model was built into these refinements.

For the data set at 960 s delay, a total of 476,372 particles were identified, with 303,375 (63.7%) contributing to a PrPfr map at 3.07 Å resolution, and 172,997 (36.3%) particles belonging to an open PfrPfr reconstruction, resolved at 3.29 Å resolution (Figure S4).

Estimation of relative concentrations at each time point was done by generating a consensus reconstruction (where applicable), then splitting the final particle set into Pr/PrPfr/Pfr groups by 3D classification in input initialization mode, supplying high-resolution (<3 Å) input maps and filtering to 4 Å.

### Spectroscopy

UV–vis spectroscopic measurements for the pH-dependent dark reversion rates were conducted under conditions as described by Prodhan et al. (2025): the protein was kept in a buffer consisting of 50 mM Tris and 50 mM NaCl at a concentration of 0.6 mg/mL, with pH ranging from 6.0 to 10.0.^38^ The temperature was set to 25 °C. The sample was kept in complete darkness or under dim green light during preparation and at the start of the measurement was illuminated with a 780 nm light source over 30 s (total dose: ∼1 mJ/mm^2^). The dark reversion was followed by collecting full UV–vis spectra (250–800 nm) at 45 s intervals with a total of 100 repeats using a Shimadzu UV-1900i spectrophotometer equipped with LabSolutions UVVis software (Version 1.13). The first spectrum was collected 5 s after far-red illumination. The absolute decay of Pr (at 698 nm) and growth of Pfr (at 749 nm) was fitted with a general exponential function

$$A\left(t\right)=B\cdot e^{-t/\tau }+C$$

with *B*, *C*, and τ as parameters to fit. The rate constant *k* is the reciprocal of τ­(*k* = τ^–1^). We then fitted the *k* vs pH plot with a sigmoidal function, i.e., a Henderson–Hasselbalch-like function

$$k\left(pH\right)=k_{low}+\frac{k_{high}-k_{low}}{1+10^{pK_{a}-pH}}$$

where *k*_low_ is the rate constant at low pH and *k*_high_ is the rate constant at high pH. From that the p*K*_a_ was directly extracted.

### Molecular Dynamics Simulations

We performed molecular dynamics simulations of both the full length PrPr and hybrid PrPfr states. Thus, the structures were protonated with H++ at pH 8 according to the experimental conditions (H277 is protonated as HIE).^61–63^ As force-field ff14SB^64^ was used as well as the TIP3P^65^ water model (10 Å minimum wall distance),^66,67^ utilizing the AMBER24 tool kit, i.e., the tleap implementation.^68^ Charges were neutralized with a uniform background charge and the systems were equilibrated with a multistep protocol.^64,69,70^

Both systems were simulated for approximately 250 ns three times (see Table 1) in an NpT ensemble (using pmemd.cuda),^71^ using a time step of 2 fs, since the SHAKE algorithm was applied.^71,72^ The particle-mesh Ewald (PME) method took care of electrostatic interactions. The langevin thermostat kept the temperature at 300 K and the Monte Carlo barostat kept pressure at 1 bar.^73,74^

**Table 1:** Summary of Simulation Frames and Time of All Repetitions

	PrPr		PrPfr	
simulation repetitions	frames	time (ns)	frames	time (ns)
1	12465	249	11901	238
2	17832	357	12500	250
3	16472	329	15271	305

The performed simulations were analyzed and visualized with cpptraj, vmd and PyMOL (The PyMOL Molecular Graphics System, Version 3.0, Schrödinger LLC).^75,76^ RMSF (root mean square fluctuation) calculations and interaction analysis were performed with cpptraj and the GetContacts tool (https://getcontacts.github.io/) and an associated python script.^77^ For the RMSF the structures were aligned on the respective GAF domain before calculation of the RMSF, which were thus subtracted from one another (scale ranges between 2 Å). To show that the assignment is reliable we calculated the difference of each protomer A to each protomer B across all PrPr simulations (Figure S11). For the hydrogen bond analysis of H277 we used the cpptraj command hbond and selected as donor H277 and as acceptor the chromophore. For the interface interaction analysis, we combined hydrogen bonds, salt bridges, and van der Waals interactions calculated with GetContacts and applied a cutoff of 0.5 for the difference between the interaction frequencies to highlight the main differences between the two geometries.

## Supplementary Material



## Data Availability

The composite cryo-EM map used to reconstruct the PrPfr model has been deposited in EMDB (EMD-53916). The PrPfr model has been deposited in the Protein Data Bank (9RCC). Raw movies and final particle sets for the time points have been deposited in EMPIAR (EMPIAR-12874). Molecular dynamics simulations have been deposited in Zenodo (10.5281/zenodo.15796800).

## Notes

Details on the process of structure refinement; plots on the molecular dynamics simulations; structural components of interest; and additional information on the pH-dependent dark reversion rate measurements (JA5C17531_SI_001)
